# The Human Gut Microbiome as a Potential Factor in Autism Spectrum Disorder

**DOI:** 10.3390/ijms23031363

**Published:** 2022-01-25

**Authors:** Amani Alharthi, Safiah Alhazmi, Najla Alburae, Ahmed Bahieldin

**Affiliations:** 1 Department of Biology, Faculty of Science, Majmaah University, Al Zulfi 11932, Saudi Arabia; 2 Department of Biological Sciences, Faculty of Science, King Abdulaziz University, Jeddah 21589, Saudi Arabia; shalhazmi@kau.edu.sa (S.A.); nalbourai@kau.edu.sa (N.A.)

**Keywords:** autism spectrum disorder, gut microbiome, gut–brain axis, epigenetics, probiotics

## Abstract

The high prevalence of gastrointestinal (GI) disorders among autism spectrum disorder (ASD) patients has prompted scientists to look into the gut microbiota as a putative trigger in ASD pathogenesis. Thus, many studies have linked the gut microbial dysbiosis that is frequently observed in ASD patients with the modulation of brain function and social behavior, but little is known about this connection and its contribution to the etiology of ASD. This present review highlights the potential role of the microbiota–gut–brain axis in autism. In particular, it focuses on how gut microbiota dysbiosis may impact gut permeability, immune function, and the microbial metabolites in autistic people. We further discuss recent findings supporting the possible role of the gut microbiome in initiating epigenetic modifications and consider the potential role of this pathway in influencing the severity of ASD. Lastly, we summarize recent updates in microbiota-targeted therapies such as probiotics, prebiotics, dietary supplements, fecal microbiota transplantation, and microbiota transfer therapy. The findings of this paper reveal new insights into possible therapeutic interventions that may be used to reduce and cure ASD-related symptoms. However, well-designed research studies using large sample sizes are still required in this area of study.

## 1. Introduction

Autism spectrum disorder (ASD) is a complicated neurodevelopmental disorder characterized by decreased verbal and social interactions, limited interests and activities, and repetitive behaviors [1,2]. Along with these significant conditions, ASD regularly co-occurs with other clinical symptoms, including gastrointestinal disturbances (up to 70%), motor deficits (79%), sleep problems (50–80%), and intellectual disability (45%) [3].

Autism prevalence has risen dramatically worldwide in the last few years, reaching 1 in 132, and with a remarkable increase in occurrence in boys compared with girls [2,4]. In the United States, the prevalence of ASD rose from 1 in 150 children in 2000 to 1 in 54 in 2016 [5]. The dramatic increase in ASD reduces parental productivity and increases the financial burden on families, with central expenditures being linked with special schooling [6].

For many years, a high number of studies have been conducted worldwide focusing on the potential etiology of ASD; however, its precise etiology has not been clearly identified. Gene and chromosomal abnormalities, such as fragile X syndrome (FXS); tuberous sclerosis (TSC); and potential defects in chromosomes 2q, 7q, 15q, and 16p, are shown in 35 to 40% of ASD cases. Furthermore, the ASD rate was found to be higher in monozygotic twins than in dizygotic twins and was found to be 50-fold higher among siblings who belong to families that already have ASD children [7]. Additionally, the multigenic disorder of autism has been related to epigenetic effects [8]; nevertheless, no specific gene has been identified as being associated with all cases of ASD. Recently, 100 to 800 genes or genomic regions have been implicated in ASD etiologies [9,10].

**Figure 1:**
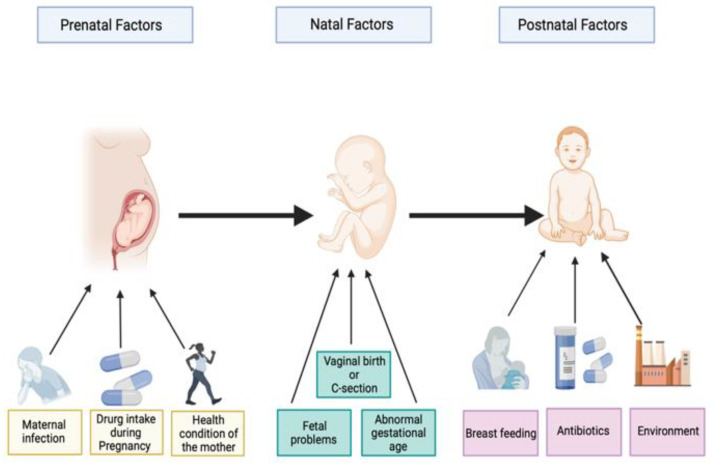
Illustration of some prenatal, perinatal, and postnatal factors associated with autism spectrum disorders. Created with BioRender.com.

Several studies have found that 60 to 65% of autism occurrence could be explained by prenatal, natal, and postnatal environmental risk factors (Figure 1). Prenatal risk factors involve maternal infection, maternal physical health, the health condition of pregnant women, folate and iron deficiency, and drug use in pregnancy. Natal risk factors include fetal complications, umbilical cord complications, hypoxia (lack of oxygen), cesarean delivery, abnormal presentation of the fetus, and abnormal gestational age (preterm or post-term). Postnatal risk factors include breastfeeding, air contamination, antibiotic intake, and nutrition factors [10,11,12,13,14]. Environmental risk factors can directly influence the neuronal activities of the growing brain of the fetus [13]. These environmental risk factors are largely found to shape the intestinal microbiota [15]. Therefore, the lack of an imprecise cause of the development of autism disorder has prompted scientists to look into other putative triggers, such as the intestinal microbiota.

The human gut comprises millions of microorganisms, and it has been suggested that a well-balanced gut microbial composition helps to maintain microbial homeostasis. At the same time, alterations in microbial composition frequently end with a negative influence on the health condition of human beings [16]. Presently, the gastrointestinal (GI) tract is considered a new organ that makes numerous metabolites and neuroactive substances. About 40% of all human metabolites are generated by the gut microbiome [17,18]. As a result, any imbalance in the community and quantity of gut microbiota during a critical time in a child’s development may impact the central nervous system (CNS) and enteric nervous system (ENS), which comprise the microbial gut–brain axis [18]. This axis describes how the gut flora can communicate with the brain and how they can impact each other [14].

Various emerging findings have revealed an alteration in the gut microbial composition in ASD individuals compared to neurotypically developing children [19]. Interestingly, GI symptoms, including abdominal pain, gastroesophageal reflux, flatulence, and constipation, have frequently been described to occur at rates of 9–84% in ASD children [20]. This avenue of analysis is essential for defining the role of microbiota dysbiosis in ASD and launching a possible treatment for ASD patients. Therefore, this paper aims to review the role of gut microbiota dysbiosis in the pathology of ASD, focusing on the microbiota–gut–brain axis. Moreover, the current review examines the present therapeutic approaches for ASD. Therefore, the review adds to our understanding of the responsibility of gut microbes in influencing ASD in humans.

The methods of this review article were based on the utilization of virtual databases, including PubMed and Science Direct, to search for all related published studies, whereas the statistics of the prevalence of ASD were taken from the website of Centers for Disease Control and Prevention (CDC). All studies included in this review were published from 2003 to 2021. The selection was based on the keywords “ASD“, “autism”, “gut” “microbiome”, “microbiota”, “gut-brain axis” “probiotics”, and “fecal transplantation”. Nearly 235 articles were found, and those examining the gut microbiota and general neurodegenerative disorders—particularly autism spectrum disorder—were included in this review article.

## 2. The Gut Microbiota

Human beings have co-evolved with a massive number of microorganisms that colonize almost every part of the body, particularly the skin, eyes, respiratory pathway, urogenital pathway, and intestine [21]. These microorganisms include bacteria, fungi, viruses, archaea, and protozoa [22]. The community of these microbes is called the microbiota, where the term microbiome indicates the genomes of these microorganisms [23]. It is believed that a series of microbial establishment events in the gut starts during the prenatal period, as proposed by the existence of microbes in the placenta, amniotic fluid, meconium, and the blood of the umbilical cord [24]. Interestingly, the significant periods of alteration in the evolving microbiota overlap partly with the timespan for the development of the brain [25]. The colonization of the newborn baby gut begins during birth—e.g., the newborn infant born through the vagina becomes covered with the mother’s vaginal microbes, or of the mother’s skin in the situation of a cesarean delivery (C-section) [26]. About 75% of the feces microbiota of vaginally born babies were found to be related to their mothers’ fecal microbiota, whereas, in C-section babies, this percentage decreases to ~41% [27].

**Figure 2:**
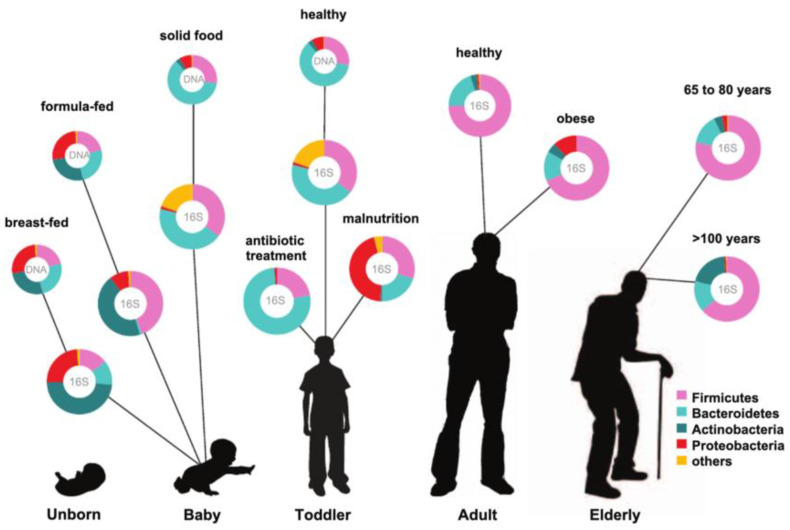
Relative abundance of the main phyla of the human gut microbiota throughout different stages of life, investigated by either metagenomic (DNA) or 16S RNA sequencing approaches. This figure is taken from Ottman et al. [35].

Following delivery, the baby is introduced to bacteria during breastfeeding, through the intake of food, and from the surrounding environment [28]. Many research studies have found that the diversity of the gut microbiota is decreased in formula-fed compared with breastfed children [29]. Key alterations in the gut microbial composition occur throughout the weaning period because the infant moves from consuming formula or breast milk to solid food [30]. In adolescence, microbial diversity and functional capacities develop toward an adult-like microbial profile, with each individual having a unique microbial community [14,31,32]. No two people have the exact same microbial community—not even monozygotic twins [33]. In adulthood, nutrition and antibiotics are the essential aspects that impact the composition of the gut microbiota across the human lifespan [25]. Consequently, the description of the adult microbiota state as ‘stable’ is somewhat imprecise, as the gut microbial community alters over time and can be restored after changes [34] (Figure 2).

Metagenomic analysis has revealed that the gut of the human body encodes about 3.3 million gut microbial composition genes, which is 150-fold higher than that of the whole human genome. Bacteria represent the most prevalent members of the intestinal microbial community, since the human body contains 1013 human bacterial cells, with a human-to-bacterial cell ratio of approximately 1:1 [36].

Several analysis approaches have recently enabled scientists to detect and quantify gut microbial components by examining nucleic acids (DNA and RNA) collected from feces. Most of these methods rely on DNA extraction and the amplification of the 16S ribosomal RNA gene (rRNA) [37]. 16S ribosomal RNA (rRNA) sequencing provides a taxonomic characterization of microbial communities. Whole-genome shotgun (WGS) (whole DNA sequencing) is able to derive the highest-quality data of functional and organismal human microbial communities [38]. Based on the available data from the Human Microbiome Project and Metagenomics of the Human Intestinal Tract (MetaHIT), about 2776 microorganisms have been extracted from human feces and categorized into 12 bacterial phyla, with *Firmicutes*, *Bacteroidetes*, *Actinobacteria*, and *Proteobacteria* accounting for up to 90% of total bacteria, while *Verrucomicrobia* and *Fusobacteria phyla* exist at a low abundance [21].

Since mammals cannot synthesize many vital nutrients, the gut microbes perform essential functions for the host, delivering necessary nutrients by digesting dietary compounds, protecting against opportunistic microbes, and contributing to the integrity of intestinal epithelial barriers [19]. Based on all these features, the complex human–microbiota interconnection can be considered an existent superorganism whose disturbance could lead to disease onset [39]. As a result, this path may help us to develop beneficial microbiota-associated therapies in the upcoming years.

## 3. Evidence Linking Gut Microbiome Dysbiosis to Autism

There are many reasons why researchers link ASD symptom development with the gut microbial composition. For example, in 2019, Sharon and colleagues reported that germ-free (GF) mice display ASD-like behavior after being colonized with fecal microbiota from children with ASD. In this study, gut microbiota were transplanted to GF mice from human donors with autism, or from their typically developing (TD) siblings as a control. Mice colonized with ASD microbiota displayed more autistic behaviors compared to the TD mice. The team also found that the ASD group had a different abundance of *Clostridiaceae*, *Lactobacillales*, *Enterobacteriaceae*, and *Bacteroides* in comparison with the TD group. They also observed that mice that were treated with gut microbiota from ASD patients displayed an alternative splicing of many ASD-related genes in the brain. Moreover, some metabolite profiles were detected to be decreased in the ASD group—particularly 5-aminovaleric acid (5AV) and taurine [40] (Table 1).

On other hand, compared to neurotypical children, ASD patients were largely found to have altered gut microbial compositions [41]. Nonetheless, no particular microbial species have been found to be consistently changed in all ASD microbial studies, which could be related to changes in different aspects such as diet, age, gender, population, and autism severity [16,42]. Although the alterations in gut microbial in ASD patients are not always consistent between studies, ASD patients frequently have microbial imbalances of many types, most notably a decreased *Bacteroidetes/Firmicutes* phyla ratio, which could be the result of a decrease in the relative abundance of *Bacteroidetes* [36]. The *Bacteroidetes* phylum is responsible for polysaccharide digestion. Therefore, this research could support the theory that ASD patients have an abnormal digestion of carbohydrates and mucosal dysbiosis in the gut [1,43]. In other studies, a high level of *Actinobacteria* phylum was noticed in autistic patients in comparison with the control group. ASD patients were also found to have a high abondance of the *Betaproteobacteria* class and high levels of *Lactobacillus*, *Ruminococcus*, and *Escherichia-Shigella* species. In contrast, the prevalence of *Bifidobacterium* and *Enterococcus* species was decreased [42,44,45]. Kang and others revealed a decline in the abundance of the genera *Coprococcus*, *Prevotella*, and unclassified *Veillonellaceae* in ASD patients [46]. In a previous study, certain *Prevotella* species (*P. ruminicola*, *P. oralis*, and *P. tanneries*) were shown to be decreased in irritable bowel syndrome (IBS) patients [36]. This study leads to the theory that ASD-related gastrointestinal symptoms are linked to a change in the microbial *balance* [46]. In other previous studies, the abundance of the genus level of *Akkermansia* was found to be low in ASD children, with the level of *Desulfovibrio* spp. being increased [44,47]. The latter bacteria are considered to be harmful, as they may exacerbate autistic behaviors and gastrointestinal problems [48]. In addition, although *Sutterella* spp. is usually uncommon in healthy microbiota compositions, an elevated abundance was found in the caecum and ileum intestinal tissues of ASD children [26]. Compared to neurotypical healthy individuals, ASD children were shown to have a significantly increased abundance of *Clostridium cluster* groups XVIII and *Clostridium bolteae* [42,43]. In addition, Alshammari and others observed a significantly higher rate of *Clostridium perfringens* in ASD children than in the controls [43,49]. *Clostridium* produces neurotoxins that exacerbate autistic symptoms and has been linked to the Childhood Autism Rating Scale (CARs), which determines the severity of ASD [41,50,51]. In addition, *C. perfringens* can produce toxins, especially the Beta2 toxin, which is associated with an increased ratio of GI abnormalities such as food poisoning and diarrhea [36]. Fisher and others found that the feces samples of ASD patients were 79% beta2 toxin, whereas they were only 38% in the control group [36].

In addition to bacterial changes, ASD patients have been found to have gut fungal dysbiosis. For example, *Candida albicans*, which produces ammonia and other toxins that are likely associated with autism-related behavior, was found in the guts of autistic children more frequently than in those of non-ASD children [51,52]. Furthermore, a significant increase in *Saccharomyces cerevisiae* was discovered in ASD individuals compared to non-autistic individuals. On the other hand, *Aspergillus versicolor* was found significantly less often in ASD patients. These alterations indicate a possible role of immune pathways in triggering ASD. *S. cerevisiae* can regulate immune function by activating TLR ligands and increasing TNF-α and IL-6 production, while *A. versicolor* has the potential to produce anti-inflammatory metabolites [53].

Although these studies clearly show modifications in the composition of the gut microbiota in ASD children, some research suggests that the variations between neurotypical and ASD children’s microbial composition could be due to the overuse of antibiotics by autistic children. Antibiotics influence gut homeostasis by targeting pathogens and commensal bacteria [14,28]. This may illustrate why the gut microbiota of children under three years old who have been given antibiotics is less diverse [14,28]. Additionally, the use of antibiotics by pregnant women is also related to a high risk of autism occurrence [14]. On the other hand, oral vancomycin treatment, a well-known antibiotic effective against *clostridia*, resulted in a considerable improvement in the symptoms of patients with ASD [36].

This area of research is still growing, so more studies with large sample sizes of autistic and neurotypical children who are not treated with antibiotics are needed in order to detect the specific function of gut microbiota dysbiosis in the onset of autism spectrum disorder.

## 4. The Microbiota–Gut–Brain Axis

**Figure 3:**
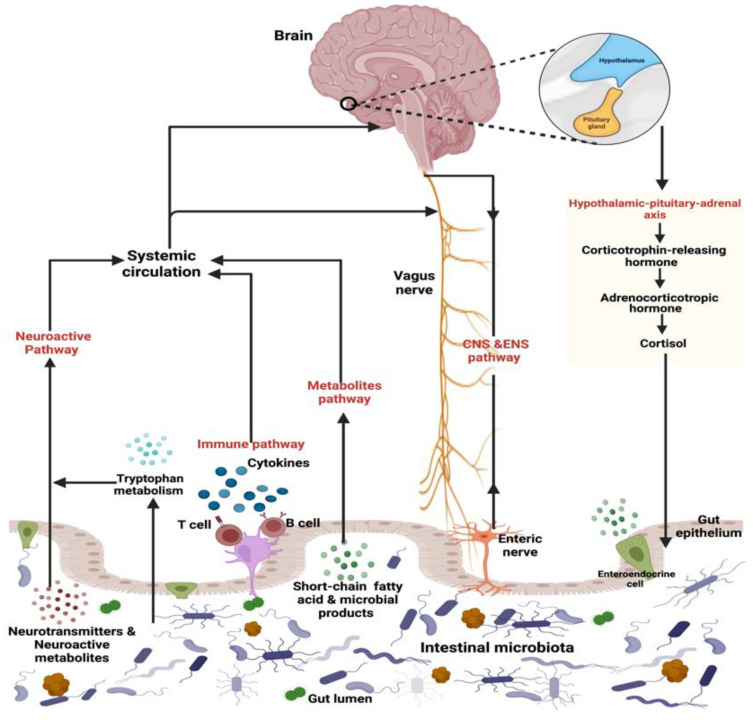
Description of the gut–microbiota–brain axis. The bidirectional communication pathways between the gut microbiota and brain are controlled by various direct (e.g., vagus nerve) and indirect paths (e.g., cytokines, short-chain fatty acids, neuroactive metabolites). Created with BioRender.com.

The gut–brain axis refers to a bidirectional connection between the gut and the brain. It can also extended to involve the microbiota as an essential part of this triangle dialogue [21]. This bidirectional pathway consists of both efferent and afferent signals. Afferent signals transmit from the gastrointestinal tract to the brain and involve the enteroendocrine system, cytokines, metabolites, gut products, and neuroactive molecules. Efferent signals start from the brain to the gut wall and include neuroendocrine and autonomic regulation [20,62]. In this pathway, 90% of vagal fibers between the brain and gut are afferent, suggesting that the intestine is more of a transmitter than a receiver [63,64,65]. This bidirectional link comprises one or more of the following avenues (Figure 3).

## 5. Signaling Pathways Based on the Gut Microbiome Composition in ASD Patients

### 5.1. Gut Permeability Pathway

The microbiota and its metabolite products modulate the function and integrity of the gut epithelium barrier. Therefore, a change in gut microbial diversity can influence the gut barrier integrity, potentially resulting in the “leaky gut” condition [66]. Indeed, an impaired gut barrier can increase the levels of gut microbial components (e.g., lipopolysaccharide (LPS)) in the blood; trigger the hypothalamic–pituitary–adrenal (HPA) axis; and stimulate immune responses, producing cytokines such as interferon-γ (IFN-γ), tumor necrosis factor-α (TNF-α), interleukin-1β (IL-1β), and IL-4. These immune cytokines can circulate and cross the blood–brain barrier (BBB), inducing systemic and CNS inflammation [67,68]. The serum level of LPS was found to be significantly increased in ASD individuals compared to healthy controls. This may be linked to a worse social communication score, which has been noticed in ASD patients [69]. In physiological states, LPS can enter the brain, possibly through a lipoprotein transport mechanism [70], and elicit neural impairment, behavioral alteration, and neuroinflammation by triggering the Nuclear Factor Kappa B (NF-kB) signaling pathway, which is related to microglia stimulation and neuronal cell loss [71]. The everyday injection of pregnant rats with lipopolysaccharide (LPS) resulted in ASD-like behavior in offspring, involving hyperlocomotion and social defects [72].

Multiple findings have suggested that ASD patients have abnormal intestinal permeabilities ranging from 43% to 76%, both with and without gastrointestinal symptoms [36]. Moreover, intestinal permeability was reported in 9 out of 21 autistic children, but not in 40 non-autistic children [26]. De Magistris and colleagues found that ASD individuals and their first-degree relatives had 36.7% and 21.2% altered gut permeabilities, respectively, while ordinary people had only 4.8% [73]. In accordance with previous studies, a significant decrease in the mRNA levels of occludin and zonulin was observed in male BTBR mice (a mouse model of idiopathic autism). Occludin and zonulin are intestinal permeability-modulating proteins that are associated with the maintenance of intestinal permeability [74,75]. Interestingly, intestinal permeability was found to be considerably reduced in autism patients who were on a gluten-free, casein-free diet [73].

In comparison with the above-mentioned studies, others have shown no changes in gut permeability in autistic children, demonstrating that the disruption of the intestinal barrier is not always a symptom of autism, but this primarily occurred in ASD children with intestinal abnormality [76,77]. Thus, additional studies with an increased sample size of ASD patients with and without intestinal abnormality are necessary to confirm and understand the connection between gut permeability and increased symptoms of autism.

### 5.2. Immune System Pathway

Immunological pathways have a vital function in the bidirectional connection between the microbiota, gut, and brain, allowing the gut and brain to influence each other. Gut microbial composition is an essential part of regulating immune hemostasis, since gut mucosal surfaces are constantly exposed to beneficial and pathogenic microorganisms and can trigger an immunological response [21,25]. In addition, the mucosal surface layers of the gut contain different types of immune cells involving gut-associated lymphoid tissue (GALT) [78]. GALT utilizes lymphocytes to produce immunoglobulins (IgA) [79]. IgA can modify the innate immune response once microbial cells come into contact with dendrites in the ENS. In some studies, a high level of IgA was recognized in ASD patients [77].

Different inflammatory signs have been found in ASD individuals. For example, elevated levels of tumor necrosis factor (TNF) and pro-inflammatory cytokines such as interferon (IFN), IL-1b, IL-6, IL-8, and IL-12p4 were found in the brains of ASD children compared to controls [80,81]. Moreover, the brains of ASD patients revealed a pattern of triggering immunological responses involving the activation of microglial cells, which are responsible for eliminating pathogens [82].

The defect in the immune system in autistic patients has been connected with the alteration of the gut microbial composition. For example, germ-free mice show a higher microglia density in various brain areas than mice grown in a specific pathogen-free (SPF) environment. Additionally, atypical social avoidance behavior and low immune response against virus infection were noticed in these GF mice. Both microglia defects and ASD-linked symptoms were improved following the supplementation of germ-free mice with microbial SCFAs [54]. This study proposed that the gut microbiota can indirectly affect the innate immune system, which can modify the circulating levels of pro-inflammatory and anti-inflammatory cytokines that directly impact microglia homeostasis.

Moreover, in the Hsiao et al. study, an increased level of IL-6 was detected in the adult offspring of a maternal immune activation (MIA) mouse model. Interestingly, the supplementation of MIA offspring with *Bacteroides fragilis NCTC 9343* restored microbiota composition, IL-6 levels, and the integrity of the intestinal permeability [55]. Several cytokines, including IL-6, were found to adjust the tight junction transcription level and intestinal barrier integrity by modulating the levels of CLDN 8 and 15. Therefore, this report proposes that the *B. fragilis*-mediated restoration of IL-6 levels might underpin the role of IL-6 in gut permeability [55].

### 5.3. The Metabolic Pathway

The gut microbiota generates various metabolites that can travel across the systemic circulation and contact the host immune cells, impact the metabolism, and/or influence the ENS and afferent signaling pathways of the vagus nerve that send signals directly to the CNS [83]. The metabolites that are derived from the microbiota include multiple products, such as short-chain fatty acids (SCFAs), phenolic compounds, and free amino acids (FAAs) [17]. Butyric acid (BA), propionic acid (PAA), and acetic acid (AA) are all types of short-chain fatty acids that result from the anaerobic fermentation of indigestible carbohydrates [84]. SCFAs play a vital function in the body such as in the homeostasis of energy, in the enhancement of glucose metabolism, in lowering body weight, and in reducing the chance of colon cancer [85]. Additionally, SCFA is implicated in the regulation of the immune response by modulating the secretion of T-cell cytokines [86].

Despite the data being slightly inconsistent, acetate and propionate have been found to be upregulated in individuals with ASD, whereas butyrate was shown to be significantly decreased [87,88]. PAA can act as a neurotoxin that affects the electron transport chain by inhibiting the formation of nicotinamide adenine dinucleotide (NADH), the primary substrate of the electron transport chain [89]. PAA can also trigger the immune response and change gene expression [14,90]. Increased levels of PAA have been related to increased severity of ASD. For example, in experimental trials, rats treated for eight days with PAA displayed hyperactivity and stereotypy movement. Additionally, PAA-treated rats exhibited significant changes in the composition of brain and plasma phospholipid molecular species. Alterations in brain plasma phospholipid composition, especially throughout development, can theoretically have severe effects on CNS function [56]. In agreement with this study, GI symptoms and modified blood phospholipid profiles have been detected in individuals with ASD. Thus, since phospholipids are the main structural components of many cellular and neuronal membranes [91], ASD, as a neurodevelopmental disorder, might be related to functional deficits or imbalances in fatty acid metabolism [92].

On the other hand, butyrate was observed to have a positive influence on ASD-related behavior [84]. In addition, butyrate can protect cells from oxidative stress and improve mitochondrial function during physiological stress [93]. Interestingly, butyrate was found to restore the ASD deficiencies introduced by PAA, likely by enhancing the BBB permeability [94]. GF mice colonized with *Clostridium tyrobutyricum* (butyrate-producing bacteria) or acetate and propionate-producing *Bacteroides thetaiotaomicron* can improve the expression of occludins, which were found to be associated with the reduced permeability of the BBB [57].

Moreover, p-Cresol and its conjugated derivatives were observed at an elevated rate in the urinary samples of children with ASD [95]. P-Cresol can aggravate ASD severity and gut function because it plays a role in many metabolic processes in the human body [87]. In addition, P-Cresol has been linked with nervous system abnormalities, including raising brain lipid peroxidation, reducing Na(+)-K+ ATPase function, and inhibiting noradrenaline formation [96]. *Clostridium difficile* is one of the most typical representative microbes and is known for forming p-Cresol. *C. difficile* can induce the p-hydroxyphenylacetate (p-HPA) enzyme and therefore stimulate the fermentation of tyrosine for the production of p-Cresol [97]. Notably, mice given p-Cresol in drinking water for four weeks exhibited an altered gut microbiota composition and social-behavioral defects [58]. The p-Cresol intervention also decreases the excitability of dopamine neurons in the ventral tegmental area (VTA) of these mice, a circuit implicated in the social reward system [98]. The influence of p-Cresol on behavior was associated with the gut microbial composition, as microbial transplantation from p-Cresol-treated mice to control mice can stimulate behavioral defects. However, microbial transplantation from normal mice to p-Cresol-treated mice was found to restore normal social behaviors [58]. This report suggested that a microbial metabolite such as p-Cresol could provoke ASD-like behavior in mice.

Collectively, all these previous studies are consistent with the emerging theory of disruption of excitatory/inhibitory neuronal function in ASD [99].

### 5.4. Neuronal Signaling Pathway

The microbiota of the gut can produce molecules such as serotonin (5-hydroxytryptamine, 5-HT), γ-aminobutyric acid (GABA), and acetylcholine, which can act as typical neurotransmitters influencing ENS and CNS activity [100]. Serotonin is one of the essential brain neurotransmitters that have a crucial function in regulating mood and GI activity [101]. About 95% of total serotonin in the human body is formed by enterochromaffin cells (Ecs) in the GI tract, while around 5% of the remaining serotonin is found in the brain [102]. Interestingly, gut microbes such as *Escherichia* spp., *Enterococcus* spp., *Streptococcus* spp., and *Candida* spp. have been shown to be engaged in the production of serotonin [103]. The production and secretion of 5-HT by Ecs have been suggested to be affected by the gut microbial composition [104]. For example, the depletion of the gut microbiota by antibiotics in mice was found to be associated with impaired learning and elevated depression-like behaviors. This occurred with changes in the levels of CNS 5-HT concentration, as well as with alterations in the mRNA levels of corticotrophin-releasing hormone receptor 1 and the glucocorticoid receptor [61]. Moreover, a positive relationship was detected between the level of 5-HT in the blood and the severity of gastrointestinal symptoms [105].

On other hand, serotonin can also be formed from the essential amino acid tryptophan (Trp) [106]. *Clostridia* spp. stimulates the transformation of tryptophan to 5-HT by raising the mRNA levels of tryptophan hydroxylase 1 in Ecs [102]. Reducing tryptophan in the diet indeed seems to increase autistic behavior. Consequently, these studies show that the gut microbiota can have a crucial role in the production and homeostasis of the 5-HT [107].

GABA is an amino acid that functions as the main inhibitory neurotransmitter in the brain. An altered pattern of GABA has been detected as a key feature of the neurophysiology of ASD patients [108]. If the inhibitory GABAergic transmission is altered in individuals with ASD, it can end in an irregular balance of excitation/inhibition in the brain and changes in neural communication, the handling of instructions, and responding performance [109]. Indeed, *Bifidobacterium* spp. and *Lactobacillus* spp. have the ability to produce GABA [110]; for example, the colonization of mice with *Lactobacillus rhamnosus JB-1* increases the level of GABA receptors in the vagus nerve and decreases stress and depressive behavior [59].

Together, these outcomes emphasize the essential function of the gut microbiota in the communication pathways between the gut microbiota and the brain, suggesting that bacteria may prove to be a beneficial treatment.

### 5.5. Neuroendocrine Signaling Pathway

The hypothalamic–pituitary–adrenal (HPA) axis is another pathway by which the brain can control the activity of intestine effector cells, gut permeability, motility, mucus, and immunity, causing the translocation of gut microbial constituents. Under stress conditions, corticotrophin-releasing hormone (CRH) is released from the hypothalamus and causes the pituitary gland to secrete adrenocorticotropic hormone (ACTH). ACTH then regulates the adrenal glands to produce and secrete hormones, such as cortisol and glucocorticoids, into the blood, which affect many bodily organs including the brain [67,100]. This initial study demonstrated that the gut microbiota can directly affect the host HPA axis. GF mice that have been exposed to restraint stress showed an increased serum concentration of the two commonly associated stress hormones ACTH and CRH. However, the colonization of young mice with *Bifidobacterium infantis* reversed hormonal abnormalities [60]. In the same study, the expression of brain-derived neurotrophic factor (BDNF) and N-methyl-D-aspartate (NMDA) receptor was also reduced in the cerebral cortex and hippocampus of GF mice, influencing the expression and release of CRH and thereby altering the HPA axis function [60]. Several studies, particularly those carried out in individuals with ASD, have found altered levels of mRNA in the glucocorticoid receptor and CRH receptor 1 [111], which basically indicates the modification of this pathway.

## 6. Role of Epigenetics in ASD

In the last few decades, the rapid rise in the rate of ASD has demonstrated that autism cannot be caused only by genetics. Therefore, scientists have examined the relationships between genetics and the environment, especially studying the role of epigenetics in causing ASD [8]. Epigenetics investigates the ways in which environmental and lifestyle factors influence DNA expression without changing the DNA sequence, which can be transmitted from one generation to another via germline cells. These epigenetic modifications can control when, or even if, a specific gene turns on and off in a cell or organism [112,113].

DNA methylation, post-transcriptional histone modifications, and gene expression regulation by non-coding RNAs are some examples of epigenetic regulation [114]. DNA methylation has been related to the etiology of nervous disorders, including ASD [115]. For example, a methylome analysis study of the human placenta exhibited a significantly higher level of a methyl group in patients with ASD through the use of pyrosequencing [116].

Several compelling pieces of evidence suggesting that the gut microbial community is directly responsible for initiating epigenetic modifications [117]. Exchange talk between microbic metabolites and external effectors such as antibiotics, nutrition, and other environmental factors can shape the epigenome (temperature, oxygen, and pH) [118]. Commensal bacteria in the gut can synthesize folate, vitamin B12, and choline, all of which are fundamental in the production of a methyl group donor (6-methyltetrahydrofolate) and the formation of S-adenosylmethionine (SAM), which is the main methyl donor in the DNA methylation process [119]. For example, *Bifidobacteria* and *Lactobacillus* species are known for folate synthesis [120]. Another critical microbial metabolite that affects epigenetics is butyric acid, a potent inhibitor of histone deacetylases [121], which removes the acetyl group from histone proteins, letting the proteins re-associate with DNA and preventing DNA transcription. Moreover, the latest suggestion shows that some endosymbiotic bacteria make small non-coding RNAs that influence host processes [122].

Based on the above-mentioned findings regarding the involvement of epigenetics in ASD, one can assume that dysbiosis in the gut microbiota composition, particularly in the early periods of development, could directly switch a specific gene on or off. In this situation, the excessive use of antibiotics may affect microbial diversity and turn on a particular gene related to autism.

## 7. The Potential Therapeutic Perspectives of ASD Targeting Gut Microbiota

**Figure 4:**
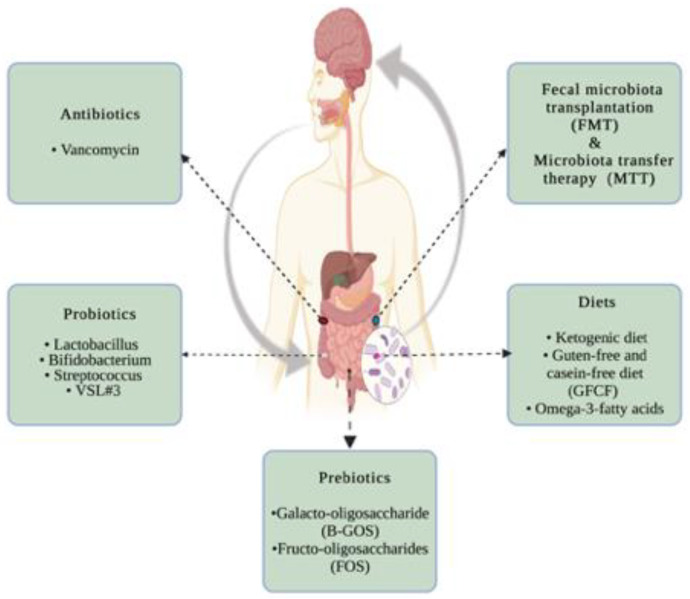
Potential therapeutic approaches for autism spectrum disorder, targeting the gut microbiota. Created with BioRender.com.

There is no current reliable therapy for treating patients with ASD. However, because of the increasing amount of data regarding the role of gut microbial dysbiosis in ASD, researchers are currently focusing on strategies for treating such a disease by modulating the gut microbial community as a potential therapeutic approach. This approach involves oral prebiotic, probiotic, dietary, and/or fecal microbiota transplantation (FMT) as well as microbiota transfer therapy (MTT; Figure 4 and Table 2).

Mounting evidence from human and animal studies suggests that gut microbial-targeting therapy may be beneficial as a new and safe method for treating ASD patients. Antibiotics have been used as a possible treatment for ASD patients, but antibiotics influence gut homeostasis by targeting pathogens and commensal bacteria. Thus, antibiotics are not a possible option for long-term therapy for ASD. Probiotics can colonize the gut and restore the composition of bacterial populations, which, in turn, has been found to reduce autism-related symptoms. Though probiotics are commonly safe to use, a study by Rondanelli et al. [131] advises that individuals with serious underlying medical illnesses or weakened immune systems should not take probiotics, since some individuals with these circumstances were found to have bacterial or fungal infections as a consequence of probiotic intake [131]. Prebiotics serve as food for commensal bacteria, so they stimulate an increase in beneficial bacteria that are found naturally in the body and improve digestive health. Studies on ASD patients using prebiotics are limited, and there is a lack of available solid data [88]. Multiple studies have shown that ketogenic diets (KD), gluten-free and casein-free (GFCF) diets, and supplementation with omega-3-fatty acids have beneficial effects on the health of children with ASD, but the evidence available is limited and weak. Marí-Bauset et al. found some possible side effects of the GFCF diet, such as calcium deficiency and a lack of essential amino acids, resulting in decreased bone density and frequent bone fractures. Moreover, ASD patients who followed the GFCF diet needed more supplementation with vitamin D [123]. Fecal microbiota transplantation (FMT) can modify the gut microbiota composition by transplanting fecal microbiota from healthy donors to ASD patients [132]. FMT has emerged as a safe and promising therapeutic approach and can restore metabolites and immune function. However, FMT could have future unexpected health effects, since there are many microbes in the gut that have not been determined yet which may introduce pathogenic bacteria into the host’s intestinal system. Therefore, to obtain the most benefit from fecal transplantation, further strict donor screening is needed to minimize the risk of FMT. Microbiota transfer therapy (MTT) was found to reduce gut and ASD-like symptoms and regulate the gut microbiota of autistic individuals [124].

### 7.1. Probiotics

Probiotics are a group of living microorganisms that are well known for improving health conditions by re-establishing the gut microbial composition [132]. Although the mechanism involved is yet to be identified, it has been reported that probiotics may lower gut inflammation by decreasing the intestinal barrier permeability and reducing the inflammation produced by cytokines and other immunomodulatory effects [133]. Grossi and others introduced a case study in which ASD patients with serous cognitive impairment were treated for four weeks with the supplementation of VSL#3 (a combined mixture of live cells of 10 different probiotics). The treatment markedly alleviated autistic symptoms and relieved the severity of gastrointestinal symptoms. Furthermore, four months of daily supplementation with three probiotics containing *Lactobacillus* strains, two *Bifidobacterium* strains, and a *Streptococcus* strain normalized the ratio of *Bacteroidetes/Firmicutes* and decreased the abundance of *Bifidobacterium* sp. and *Desulfovibrio* spp. in the feces of autistic children [125]. Additionally, probiotic supplementation significantly reduced levels of TNFα. This study suggests that probiotic supplementation alters the gut microbial composition in ASD children [44]. Another study reported lower amounts of D-arabinitol in the urine of ASD children who received oral supplementation with an *L. acidophilus* strain, and it enhanced their ability to follow instructions [134]. These studies assumed that the appropriate use of probiotics could reduce autism-related symptoms, but additional studies are needed.

### 7.2. Prebiotics

Prebiotics are non-digestible oligosaccharides that stimulate an increase in beneficial bacteria found naturally in the body, especially *lactobacilli* and bifidobacteria. In general, the bacterial fermentation of prebiotics produces SCFAs, which are linked to their health benefits [121,135]. In an in vitro study on a gut model, the analysis of feces samples from children with ASD and non-autistic children showed that the prebiotic Galacto-oligosaccharide (B-GOS) raises the abundance of *Bifidobacterium* spp. [88]. Although probiotic treatments have been shown to relieve GI symptoms and regulate the gut microbiota, studies on ASD patients using prebiotics are limited and there is a lack of available solid data [88,104].

### 7.3. Dietary

According to the findings of many studies, autistic children strongly prefer starchy foods, snacks, and processed foods. Additionally, they consume fewer fruits, vegetables, and proteins than typical non-autistic children [136]. In addition, it is recognized that most ASD children are underweight because they ingest lower daily levels of vitamins, dietary fibers, calcium, and potassium [41]. In both human and animal models, research has demonstrated that ketogenic diets (KD) have some potential positive effects on the performance and symptoms of autistic patients. KD with a high-fat content (65–90%) is commonly used to lower ASD symptoms [41]. Other than KD, vitamins, minerals, omega-3-fatty acids, and antioxidants are thought to have beneficial effects for ASD. For example, the treatment of ASD patients with omega-3 fatty acids for 12 weeks enhanced their social behavior dramatically [126]. Multiple studies have shown that a gluten-free and casein-free (GFCF) diet is beneficial for the health of children with ASD [41]. However, in 2015, a study found that a GFCF diet plan had side effects due to calcium deficiency and a lack of essential amino acids, resulting in decreased bone density and frequent bone fractures. Moreover, ASD patients who followed a GFCF diet were found to need more vitamin D supplementation [123].

### 7.4. Fecal Microbiota Transplantation (FMT)

Fecal microbiota transplantation (FMT) modifies the gut microbiota composition by fecal transplantation from healthy donors to the patient [137]. FMT was developed to treat inflammatory bowel disease (IBD) and irritable bowel syndrome (IBS) patients based on the theory that it could help with constipation symptoms [138,139]. As a result, researchers are keen to investigate the use of FMT to cure ASD children. However, because some adverse effects, such as diarrhea, abdominal pain, bloating, and transitory low-grade fever have been recorded, the safety of FMT should be considered further [140].

### 7.5. Microbiota Transfer Therapy (MTT)

Microbiota transfer therapy (MTT) is similar to FMT. Nevertheless, MTT involves two weeks of antibiotic treatment, a bowel cleanse, a stomach acid suppressant, and a fecal microbiota transplant with a high starting dose for 7–8 weeks. MTT has been found to reduce gut and ASD-related symptoms and regulate the gut microbiota of autistic individuals [124].

## 8. Conclusions and Future Directions

The increased rate of ASD shows an urgent need to detect the etiology and pathogenesis of autism. In the last few decades, accumulating evidence has implicated gut microbial dysbiosis in the etiology of ASD, as it has an essential role in various important body functions involving the development of the central nervous system (CNS) and neuropsychological homeostasis, in addition to the health of the gastrointestinal (GI) tract. There are several pathways by which the microbiota of the gut or their components affect the brain. A deeper understanding of these pathways could open up novel avenues that allow the beneficial treatment of ASD patients, reducing ASD-related symptoms and improving patients’ quality of life.

Even though gut microbial dysbiosis has been linked to ASD pathogenesis, at present, it is not likely to define a single microbe as a hallmark of ASD. This is due to the lack of consistent analysis approaches as well as the heterogeneity of enrolled participants—including participants’ age and sex, the different scales used for the evaluation of ASD symptoms, the presence/absence of gastrointestinal symptoms, and the different dietary lifestyles followed. Moreover, most of the reports enrolled a small number of ASD individuals who do not represent most of the ASD population. However, in future studies of FMT, the use of large sample sizes may lead to the identification of definite combinations of beneficial microbes that can be used to cure ASD.

To deeply examine the role of intestinal microbes in ASD, additional studies should focus on another unexplored area that can help identify the distinctive ASD microbiome. For example, current studies have mostly explained changes in gut bacteria—only a few studies have focused on fungi, and no studies have been carried out on other gut microbiota, such as protozoa, viruses, and archaea. Likewise, the application of multi-omics approaches in future research is highly recommended in order to gain more conclusive outcomes.

## Figures and Tables

**Table 1:** Animal models linking gut microbiota dysbiosis to ASD.

Animal Model	Behavior	Major Finding	Ref
GF mice	Social behavior and repetitive behaviors	-GF mice were transplanted with microbiota from humans with ASD or TD siblings. -GF mice colonization with ASD microbiota, but not TD, display autistic-like behaviors. -Alternative splicing of many ASD-related genes was found in the brain of ASD mice. -ASD colonized mice have a different abundance of *Clostridiaceae, Lactobacillales*, *Enterobacteriaceae*, and *Bacteroides*.	[40]
GF mice	Impaired innate immune system	-GF mice were orally supplemented with microbial SCFAs. -SCFAs regulate the impaired microglia maturation observed in GF mice.	[54]
MIA mouse	ASD-like behaviors	-MIA mice offspring demonstrated disruption in the gut barrier, elevated IL-6 levels, and decreased cytokine/chemokine levels. -About 8% of gut microbial metabolites in MIA offspring were altered. -MIA offspring exhibited autism-related behaviors. -MIA offspring were orally treated with Bacteroides fragilis for six days at the weaning stage. B. fragilis was found to regulate gut permeability, restore microbial composition, reduce ASD-like defects, and restore IL-6 levels.	[55]
Rats	ASD-like behaviors	-Rats were injected with PPA. -PAA-treated rats induced abnormal ASD-like behavior and increased locomotor activity. -PAA rats significantly exhibited changes in the brain composition and the plasma phospholipid’s molecular species.	[56]
GF mice	Increased BBB permeability	-GF adult mice were colonized with either butyrate-producing bacteria *Clostridium tyrobutyricum* or *Bacteroides thetaiotaomicron*, which primarily produce acetate and propionate. -Exposure of GF adult mice to *C. tyrobutyricum* or *B. thetaiotaomicron* enhanced the integrity of the BBB and upregulated the transcription of tight-junction occludin and claudin-5 proteins.	[57]
Mice	ASD-like behaviors	-Mice were treated with p-Cresol in drinking water. -p-Cresol mice presented stereotypies and abnormal social behaviors which were linked with a decline in activity of central dopamine neurons. -Transplantation of microbiota from p-Cresol- mice to untreated mice revealed increased fecal p-Cresol concentration and induced social deficits. -Colonization of p-Cresol with microbiota from untreated mice was found to restore social interaction deficits, dopamine neuron excitability, and fecal p-Cresol levels.	[58]
Mice	Anxiety and depression-linked behaviors	-Stress model mice were orally were treated with *L. rhamnosus (JB-1).* -*L. rhamnosus* probiotic induces activation of GABA receptors and decreases stress	[59]
GF mice	Stress response	-GF mice received gut microbiota by fecal transplantation from SPF animals. -GF mice exposed to restraint stress displayed a high secretion of ACTH and CRH and had a reduced expression of BDNF in the cerebral cortex and hippocampus. -GF treated with *Bifidobacterium infantis* showed a reversal in stress hormonal abnormalities, whereas microbiota from SPF partially restored hormonal irregularities in GF mice, but only if this was carried out early in life.	[60]
Sprague Dawley rats	Depressive-like behaviors	-Ten-week-old Sprague Dawley rats were treated with antibiotics. -Alteration in CNS serotonin levels. -Antibiotic treatment throughout adulthood leads to deficits in spatial memory. -Increased incidence of depressive-like behaviors.	[61]

GF—germ-free mice; TD—typically developing; MIA—maternal immune activation; PAA—propionic acid; BBB—blood–brain barrier; GABA—γ-aminobutyric acid; CRH—corticotrophin-releasing hormone; ACTH—adrenocorticotropic hormone; BDNF— brain-derived neurotrophic factor; SPF—specific pathogen-free.

**Table 2:** Summary of interventional studies modifying the gut microbiota to reduce gastrointestinal symptoms in ASD patients. SRS—social responsiveness scale; ABC—aberrant behavior checklist; CGI—clinical global impression.

Subject	Intervention	Protocol	Key Finding	Ref
-10 ASD children (2–9 years old) -9 of their siblings (5–7 years old) -10 control group (2–11 years old)	Probiotic	-The participants received one capsule three times a day for four months. This capsule contained three *Lactobacillus* strains, two *Bifidumbacteria* strains, and one *Streptococcus* strain, with percentages of 60, 25, and 15%, respectively.	In ASD participants, probiotic supplementation normalized the ratio of the *Bacteroidetes/Firmicutes*, decreased the abundance of *Desulfovibrio* spp. and *Bifidobacterium* spp., and significantly reduced the levels of TNFα.	[44]
-3 Autistic children -3 Non-ASD children (5–10 years old, male)	Prebiotic	-Galactooligosaccharide (B-GOS) was applied in an in vitro gut model system.	Prebiotic treatment elevated the abundance of *Bifidobacterium* spp. and increased acetate and butyrate fatty acids.	[88]
-105 ASD patients aged 6–9 years old	GFCF diet	-20 members of the study followed a gluten-free, casein-free diet for at least three months, while the remaining 85 participants were on a regular diet.	GFCF intervention led to decreased weight, body mass index (BMI), total energy, calcium, vitamin B5, phosphorus, and sodium consumption, but an increased intake of legumes, fiber, and vegetables. Moreover, the other group who followed the GFCF diet needed more vitamin D supplementation.	[123]
-18 ASD children with GI-moderated symptoms aged 7–16 years old	Antibiotic + Microbiota Transfer Therapy (MTT)	-For 14 days, oral vancomycin was given to the participants, and on the 12th day of vancomycin, children received Prilosec. Then, the participators fasted for 12–24 h with bowel cleansing. After fasting, participants underwent eight weeks of microbiota transplant therapy from healthy donors.	At the end of the intervention plan, the GI symptoms were reduced by 80%, and there were significant improvements in the ASD core symptoms. In addition, beneficial shifts in the composition of the gut microbiota were also seen after the therapy. These improvements extended 8 weeks after the end of the intervention.	[124]
Twelve-years-old ASD boy	Probiotic	-The ASD child was given VSL#3 (a mixture of ten live strains of *Bifidobacteria*, *lactobacilli*, and Streptococci). The probiotic treatment lasted for four weeks, followed up by a four-month treatment.	The probiotic intervention lowered the GI symptom severity and reduced ASD-related symptoms.	[125]
-41 ASD volunteers aged 7–18 years old	Omega-3 fatty acids supple-mentation	-Participants were given omega-3 fatty acids for twelve weeks.	The omega-3 intervention significantly improved the core symptoms of ASD and attention problems and altered the fatty acid profile.	[126]
-35 people with ASD aged from 3 to 20 years old	Probiotic	-The members of the study were randomly divided into two groups. -The first group received daily *Lactobacillus Plantarum*. -The second group received a placebo. -Both groups were treated for twenty-eight weeks. -After fifteen weeks, both groups were given oxytocin.	Probiotics and oxytocin intervention improved ABC, SRS, and CGI scores. Additionally, the combined treatment positively changed the gut microbiome composition.	[127]
-26 children with ASD aged 3 to 9 years old	Probiotic and prebiotic	-ASD participants were separated into two groups. The first group had 16 participants and was given FOS (fructo-oligosaccharides), while the second group had 10 children who received a placebo. -Both groups received one pack of a probiotic mixture containing 1010CFU (*B. lactis* BL-04, *L. rhamnosus* HN001, *B. infantis Bi-26*, and L. paracaseiLPC-37) per day for 30–108 days.	No alterations were seen in the group that received a placebo. However, the other group had a significant decrease in GI symptoms and ASD severity. Moreover, the FOS group was found to have an increased level of beneficial microbes such as (*Bifidobacteriales* and *B. longum*). In addition, FOS + probiotic was found to suppress the abundance of *Clostridium*.	[128]
-85 ASD participants aged between 18 and 72 months (55 without GI symptoms and 30 with GI symptoms) -Only 63 children completed the trial	Probiotic	-Participants were randomly distributed. -In the first month of treatment, 42 participants received two packets per day of probiotic. The same group was given one pack of De Simone Formulation per day in the following five months. Every package contained 450 billion *S. thermophilus*, *B. breve*, *B. longum*, *B. infantis*, *L. acidophilus*, *L. plantarum*, *L. paracasei,* and *L. delbrueckii subsp.bulgaricus.* -43 participants received placebo packets, including 4.4 g of maltose and silicon dioxide, for a four-month experimental trial.	Participants with gastrointestinal symptoms who completed the study and received the probiotic treatment were found to show an improvement in some gastrointestinal symptoms, sensory profiles, and adaptive functioning compared to the other group who were given a placebo.	[129]
-30 children with ASD aged 4–11 years old	(B-GOS) prebiotic + (GFCF) diet	-Participants were split into two groups, A and B. Four subjects subsequently dropped out, and only 26 participants completed the ten-week study. -Group A was on an unrestricted diet; out of 14 participants within this group, 7 children received a placebo and the other 7 received B-GOS. -Group B had 12 participants who were on a restricted diet (GFCF; 6 participants received a placebo, and 6 participants received B-GOS).	Children following GFCF diets had significantly lower abdominal pain and bowel movement scores. Following a restricted dietary approach also resulted in lowering the abundance of *Bifidobacterium* spp. And the *Veillonellaceae* family. The combined intervention of GFCF and prebiotic resulted in improvements in antisocial behavior.	[130]

## Data Availability

No new data were created or analyzed in this study. Data sharing is not applicable to this article.
